# Immunization With *Mycobacterium tuberculosis* Antigens Encapsulated in Phosphatidylserine Liposomes Improves Protection Afforded by BCG

**DOI:** 10.3389/fimmu.2019.01349

**Published:** 2019-06-12

**Authors:** Gil R. Diogo, Peter Hart, Alastair Copland, Mi-Young Kim, Andy C. Tran, Noemi Poerio, Mahavir Singh, Matthew J. Paul, Maurizio Fraziano, Rajko Reljic

**Affiliations:** ^1^Institute for Infection and Immunity, St. George's University of London, London, United Kingdom; ^2^Dipartimento di Biologia, University of Rome Tor Vergata, Rome, Italy; ^3^LIONEX GmbH, Brunswick, Germany

**Keywords:** liposomes, tuberculosis, vaccine, BCG, immunity

## Abstract

Liposomes have been long considered as a vaccine delivery system but this technology remains to be fully utilized. Here, we describe a novel liposome-based subunit vaccine formulation for tuberculosis (TB) based on phosphatidylserine encapsulating two prominent TB antigens, Ag85B, and ESAT-6. We show that the resulting liposomes (Lipo-AE) are stable upon storage and can be readily taken up by antigen presenting cells and that their antigenic cargo is delivered and processed within endosomal cell compartments. The Lipo-AE vaccine formulation combined with the PolyIC adjuvant induced a mixed Th1/Th17-Th2 immune response to Ag85B but only a weak response to ESAT-6. An immunization regimen based on systemic delivery followed by mucosal boost with Lipo-AE resulted in the accumulation of resident memory T cells in the lungs. Most importantly though, when Lipo-AE vaccine candidate was administered to BCG-immunized mice subsequently challenged with low dose aerosol *Mycobacterium tuberculosis*, we observed a significant reduction of the bacterial load in the lungs and spleen compared to BCG alone. We therefore conclude that the immunization with mycobacterial antigens delivered by phosphatidylserine based liposomes in combination with Poly:IC adjuvant may represent a novel BCG boosting vaccination strategy.

## Introduction

Despite the availability of a vaccine and drug regimens, tuberculosis (TB) remains a major health burden globally. In 2016, there were ~1.3 million deaths from TB among HIV-negative and 374,000 among HIV-positive individuals, making it the 9th cause of death worldwide and the number one due to a single infectious agent (1).

The current vaccine, Bacille Calmette Guérin (BCG), was first administered nearly 100 years ago and is still extensively used today. BCG was first used in children in 1921 and is now given to more than 120 million people worldwide every year, with 4 billion people already immunized (2). It is effective at preventing severe forms of TB in children but provides varying levels of protection against pulmonary TB in adults. Revaccination with BCG has not been shown to be advantageous (3, 4) and is not recommended by the WHO. Coupled with the emergence of multi-drug resistant tuberculosis (MDR-TB) with unsatisfactory treatment rates, it is clear that a more effective TB vaccine is a major healthcare priority.

Although there are several vaccine candidates at various stages of clinical trials and many more at preclinical stage of research and development, it is important to continuously feed the TB vaccine pipeline with both live and subunit vaccines. This is because it may well be that more than a single vaccination strategy will be needed to protect different human populations against TB (e.g., different age groups, HIV status, geographical location etc.). For example, a replacement BCG vaccine is likely to be another live attenuated organism (recombinant BCG or attenuated *Mtb*) in order to protect young children from severe primary TB infection but a subunit vaccine may be preferable in HIV-positive population due to the host's immunocompromised state. Likewise, it may be preferable to boost BCG with a subunit rather than another attenuated vaccine to avoid excessive delayed hypersensitivity reactions (Koch's phenomenon). Recombinant protein subunit vaccines are inherently safer but are often weakly immunogenic and require adjuvants and/or specialized delivery systems to induce protective immunity.

One such delivery system is liposomes, which were identified as a potential drug delivery platform in the 1970s. However, the discovery that liposomes preferentially target tissue macrophages (5) highlighted their potential also as a vaccine delivery system (6, 7). Subsequently, they have been used in the context of both bacterial (8, 9) and viral infections (10) and even in cancer immunotherapies (11). An attractive characteristic of the liposomal delivery platforms is that they can shelter antigens from degradation, promote phagocytosis by antigen-presenting cells (APCs) (7) and induce phagosome-cytosol cross-presentation pathway of antigenic peptides on MHC Class I molecules (12, 13).

**Figure 1:**
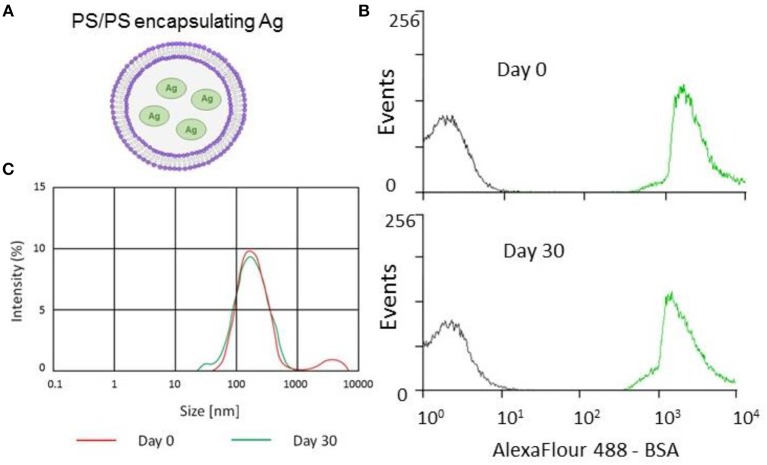
Encapsulation and stability of PS/PS liposomes and their physical characteristics. The stability of PS liposomes carrying Alexafluor488-BSA was assessed for fluorescence intensity by flow cytometry and size distribution by Malverin Zetasizer, immediately and after 30 days from liposome generation and storage at 4°C. **(A)** Schematic showing PS/PS liposome encapsulating an antigen. **(B)** Overlay of flow cytometry histogram plots of AF488-BSA loaded liposomes with “empty” liposomes over time. Gray corresponds to empty liposomes while green corresponds to AF488-BSA liposomes after 30 days storage at 4°C. **(C)** Size distribution measured by Zetasizer of Ag85B-loaded liposomes following 30 day storage at 4°C.

The apoptotic body-like liposomes (ABLs, Figure 1A) used in the present study are prepared from phosphatidylserine (PS), rendering them similar to apoptotic bodies. PS is hydrophilic and appears on the surface of early apoptotic cells (14); this “flags up” the dying cells as a target for APCs such as macrophages and dendritic cells (DC) (15, 16). It is thought that this phenomenon can augment the presentation of the antigens by APCs, leading to improved T cell responses (17). As a cell initiates apoptosis, the PS normally situated on the inner face of the lipid bilayer of the cell membrane is exposed (14). PS is implicated in the detection of apoptotic cells by its interaction with Tim4 and Tim1 on APC, which facilitates phagocytosis (18).

We therefore hypothesized that the PS associated antigens will similarly be taken up by professional APCs resulting in their cross-presentation and amplification of the immune response. Here, we present the evidence for the utility of this vaccine delivery system by demonstration that liposomally encapsulated *Mtb* antigens can enhance BCG-afforded protection against tuberculosis infection in mice.

## Materials and Methods

### Generation of PS Liposomes

Briefly, the inner monolayer lipid was prepared by suspending phosphatidylserine (PS, Avanti Polar Lipids) in 1 ml of anhydrous dodecane at the concentration of 0.2 mg/ml, sonication for 30 min and overnight incubation. The following day, 10 μg of Ag85B, or ESAT-6 (Lionex Diagnostics and Therapeutics), or bovine serum albumin conjugated to Alexafluor488 (AF488-BSA), Alexafluor647 (AF647) (Molecular Probes) or ovalbumin conjugated to BODIPY^®^ FL [500 μg/ml] (DQ-OVA), were added to inner monolayer lipid suspension and sonicated until a homogeneous solution was obtained. The inner monolayer lipid for empty control liposomes was prepared using the same buffer used to suspend the respective antigen. The outer lipid monolayer was prepared by suspending PS in 99:1 dodecane: silicone solution to get a lipid concentration of 0.05 mg/ml. Thereafter, 2 ml of outer monolayer lipid suspension was added to 3 ml of 0.9% NaCl solution. Finally, the inner monolayer lipid suspension was added to 2 ml lipid phase and the sample was centrifuged at 120 × g for 10 min. After the centrifugation, liposomes were collected in the aqueous phase using a 5 ml syringe with a 16-gauge stainless steel needle. Liposomes were quantified and characterized in terms of dimensions as described (19). To determine antigen recovery following encapsulation, liposomes were dialysed against a 100 kDa membrane (Float-A-Lyzer® G2, Spectrum Labs), according to manufacturer's instructions. Encapsulated antigen was then quantified by CBQCA Protein Quantitation Kit (C-6667 Molecular Probes) according to manufacturer's instructions by fluorimetric analysis (Thermofisher VARIOSKAN LUX). The schematic representation of liposomes with encapsulated antigen is shown in Figure 1A. For the final vaccine formulation, equal numbers of Ag85B and ESAT-6 encapsulating liposomes were combined to generate the Lipo-AE vaccine candidate.

### Generation of Human DC

Human blood monocytes from healthy volunteers were separated from peripheral blood mononuclear cells (PBMCs), by using anti-CD14 monoclonal antibodies conjugated to magnetic microbeads (Miltenyi Biotec), according to manufacturer's instructions. To obtain immature dendritic cells (iDc), cells were suspended in complete medium (RPMI 1640 supplemented with 10% fetal bovine serum, 2 mM L-Glutamine and 5 μg/ml Gentamicin) and incubated for 5 days in 24-well plates at the concentration of 5 ×10^5^ cells/well in the presence of 20 ng/mL GM-CSF (Sigma-Aldrich) and 20 ng/mL IL-4 (Miltenyi Biotec). To obtain mature dendritic cells (mDC), iDCs were further stimulated for 18 h with 100 ng/ml lipopolysaccharides (Sigma-Aldrich).

### Flow Cytometry Analysis of Stability and Antigen Delivery by PS Liposomes

The stability of PS liposomes loaded with AF488-BSA was assessed in terms of fluorescence intensity immediately and at 30 days after their preparation by flow cytometry analysis. Antigen internalization was analyzed after incubation of immature (iDC) or mature DC (mDC) with PS liposomes loaded with bovine serum albumin conjugated to Alexafluor647 (BSA-AF647) (Molecular Probes), at the ratio liposome: cell of 5:1, for 1 h at 37°C. Antigen processing was evaluated in iDC after exposure to ovalbumin conjugated to BODIPY® FL (DQ-OVA), a self-quenched conjugate that exhibits bright green fluorescence upon proteolytic processing and red fluorescence upon accumulation of proteolysed fragments in endosomal compartments. The analyses were performed by a FACSCalibur flow cytometer (Becton Dickinson).

### Confocal Microscopy Analysis of Antigen Processing in Dendritic Cells

Briefly, iDC were stained with the nucleic acid stain Hoechst (Molecular Probes) and the acidophilic dye Lysotraker Red (Molecular Probes) for 15 min at 37°C. Thereafter, cells were washed with PBS, and exposed to PS liposomes loaded with AF488-BSA at the ratio 5:1 (liposome:cell) for 90 min at 37°C. The analysis was performed by a confocal laser scanning microscope IX 81 and OLYMPUS FV1000 operating system.

### Animals

All animals were used with approval from St. George's University of London Ethics Committee under an approved UK Home Office animal project license and used in accordance with the Animals (Scientific Procedures) Act 1986. 8–10 weeks old female C57BL/6 mice were used for this study and were obtained from Charles River, UK. Animal work was conducted at St. George's University of London Biological Research Facility in accordance with local guidelines, including approval from the St. George's University of London Research Ethics Committee and national legislation, the Animals in Scientific Procedures Act, 1986. All procedures were performed under the approved UK Home Office animal project license.

### Recombinant Proteins

Ag85B and ESAT-6 antigens of *Mtb* were produced in *E. coli* by Lionex company (Braunschweig, Germany) using the standard cloning techniques and the IPTG-inducible expression vector pLEXWO481. Recombinant proteins were isolated from inclusion bodies after denaturation in 8 M urea using metal chelate chromatography (Ni-NTA Superflow, Qiagen) and subsequent refolding by dialysis. Purity was assessed by SDS-PAGE (>97% purity) and identity confirmed by Western blots specific for antigens. Endotoxin content was measured by LAL assay and determined to be <5 I.U./mg.

### Immunizations

Mice were first immunized subcutaneously at the base of the tail with 5 ×10^5^ CFU BCG Pasteur in 0.1 ml or a matched volume of phosphate saline solution (Sigma) as a control. Ten weeks later, mice received 0.1 ml of Lipo-AE formulation (containing ~10^6^ liposomes, 1 μg of Ag85B and 0.2 μg ESAT-6) also by s.c. injection at the base of the tail and then 3 weeks later a further intranasal inoculation of 0.05 ml of the same formulation, while under light anesthesia. All Lipo-AE immunizations included 20 μg/per dose of poly(I:C) as the adjuvant. Further details of the dosing regimen are given in the corresponding figure legends.

### Low-Dose Aerosol Mtb Infection and Bacterial Enumeration

Mice were infected 4 weeks after the final immunization by aerosol with *Mtb* (H37Rv strain) at a low dose of ~200 bacilli per animal. The dose was delivered by nose only exposure using the Biaera aerosol generator controlled by the AeroMP software (Biaera Technologies) and housed in a dedicated Containment Level 3 (CL3) laboratory at Biological Research facilities at St. George's. Mice were left infected for 4 weeks before culling and bacterial enumeration in the lungs and spleens. The organs were homogenized in 3 ml of 0.1% Triton x-100 using the Precellys equipment. Serial dilutions of the homogenate were prepared and plated on Middlebrook 7H11 plates supplemented by OADC (Becton Dickinson) and bacterial colonies counted 3 weeks later. The pathogenic *Mtb in vitro* work was performed in the CL3 TB suite at the Institute for Infection and Immunity at St. George's.

### Mucosal Antibody Responses

IgG and IgA in bronchoalveolar lavage (BAL) specific for antigens was measured by ELISA. Antigens (Ag85B or ESAT-6, Lionex) at 2 μg/ml were used to coat the wells of an ELISA plate. After washing with PBS 0.05% v/v Tween-20 and blocking with PBS 1% w/v BSA, 0.05% v/v Tween-20, samples were added in 3-fold dilutions. Specific IgG and IgA were detected with alkaline phosphatase conjugated anti-mouse IgG (Jackson Immunoresearch) and anti-mouse IgA (Sigma), respectively, and the substrate SigmaFast p-nitrophenyl phosphate (Sigma). Triplicate assays were read at 450 nm on a Tecan200 plate reader and data plotted as relative antibody titers as described in Hart et al. (20).

### T Cell Proliferation, Cytokine Production, and Lung Trm

T cell proliferation was assessed by measuring the incorporation of radio-labeled thymidine [^3^H] after stimulation of splenocytes with recall antigens. Erythrocyte-depleted splenocytes were seeded at 1.5 ×10^5^/well in complete RPMI 1640 medium supplemented with 10% FBS and stimulated with 5 μg/ml antigen or 1 μg.ml ConA as the positive control. After 48 h incubation, 1 μiCi of ^3^H-thymidine (PerkinElmer, Wallac, UK) was added before a further 24 h incubation. Cells were harvested with the Harvester 96 (TomTec Life Sciences, Hamdem, USA) onto Printed Filtermat paper (PerkinElmer) prior to the addition of melted wax (MeltiLex TM A. PerkinElmer) onto each scintillation sheet. Radioactive counts per minute (cpm) were measured using the 1,450 Microbeta Plus-Liquid Scintillation Counter (PerkinElmer).

For phenotypic analysis of proliferating T cells, splenocytes were stimulated with 5 μg/mL Ag85B or 1 μg/mL α-CD3 (Biolegend) for 5 days, followed by surface staining with CD4-PerCP/Cy5.5, CD8-Brilliant Violet 510, CD44-FITC, CD62L-PE, and CD90.2-Brilliant Violet 421—all from Biolegend. Cells were then fixed and permeabilized using the eBioscience Foxp3/Transcription Factor Staining Buffer Set and stained with Ki67-APC. Example of gating strategy is shown in Supplemental Figure S1.

Culture supernatant cytokine levels were measured using the mouse LegendPlexTM kit (Biolegend) according to the manufacturer's instructions. Samples were acquired on a BD FACSCalibur, and data analyzed using the proprietary data analysis software (Biolegend).

For detection of lung resident memory T cells (Trm), lungs were perfused of blood by flushing PBS through the right ventricle. Tissue was then dissected into 1 mm pieces using a scalpel, followed by digestion in 1 mg/mL collagenase and 0.5 mg/mL DNase I (Roche). Cells were then passed through a 70 μm strainer (Becton Dickinson), contaminating erythrocytes were lysed, and mononuclear cells were stained for CD3-APC, CD4-PerCP/Cy5.5, CD8-Brilliant Violet 510, CD44-FITC, CD62L-PE, CD69-PE/Cy7, and CD103-Brilliant Violet 421—all from Biolegend. Gating strategy as in Hart et al. (20).

### Sample Size, Data Presentation, and Statistical Analysis

For animal experiments, sample size calculations for *Mtb* challenge studies were based on anticipated magnitude of vaccine effect as 1 Log_10_ reduction of CFU, the intragroup variability of 0.5 Log_10_ and a confidence level of 95%. This necessitated 6 mice per group but due to protracted nature of experiments seven were used per group. For immunological evaluation three mice per group were used and data expressed as arithmetic means ± standard error. Two *in vivo* experiments were performed with similar outcomes with the dataset from one experiment shown in full in Figures 3–7, and only *Mtb* challenge data from the second experiment shown in the Supplemental Information. For all experiments based on multiple test groups, One-way ANOVA was performed followed by Dunnett's multiple comparison test. Further details of statistical analyses are described in the relevant figure legends. All analysis was performed using FlowJo v10, Microsoft Excel 2010 and GraphPad Prism 7.

## Results

### Biophysical Characterization and Uptake Analysis of PS Liposomes

Stability is an important issue to be addressed during the generation of novel liposome based vaccine formulations in order to ensure efficient antigen delivery (21). This issue has been addressed by monitoring the kinetics of the encapsulation of fluorescently labeled AF488-BSA, used as a model antigen. The analysis performed by flow cytometry and illustrated in Figure 1B shows that liposomes with encapsulated AF488-BSA remain stably fluorescent up to 30 days upon generation and storage at 4°C. Then, we encapsulated PS liposomes with mycobacterial antigens (Ag85B and ESAT-6) and further tested the physical properties of the novel liposome formulation in terms of size distribution by a Malverin Zetasizer. Results shown in Figure 1C indicate that the formulation was largely homogenous with an average size of the liposomes ~240 nm.

**Figure 2:**
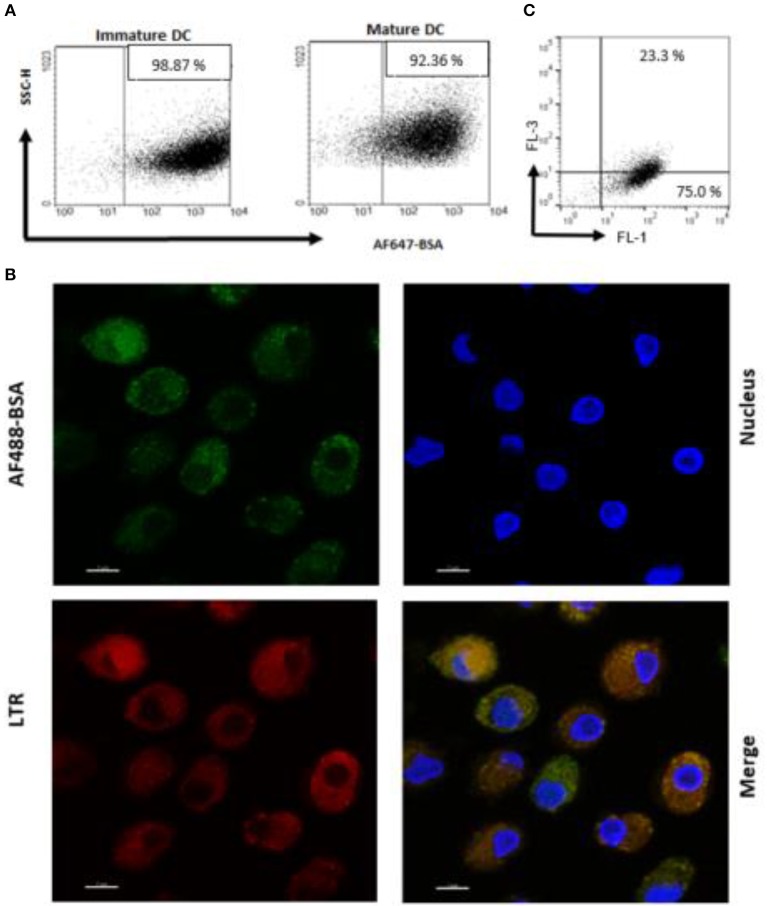
Antigen internalization and processing in human DC after delivery by PS liposome**. (A)** Immature and mature DC cells were exposed to dialyzed PS liposomes loaded with AF647-BSA to a ratio of 5:1 for 1 h. Analysis was performed by flow cytometry. **(B)** Immature DC cells were stained with the nucleic acid stain Hoechst and the acidophilic dye Lysotraker Red and stimulated with PS liposomes loaded withAF647-BSA at the ratio 5:1 for 90 min. A representative image from over many taken by confocal microscopy, is shown. Sample analysis indicated majority of cells positively stained with ~23% strongly positive for both stains. **(C)** Immature DC cells were exposed to PS liposomes loaded with DQ-OVA or to empty liposomes at a ratio of 5:1 for 1 h. Red and green fluorescence was evaluated by flow cytometry. A representative experiment with cells from one, out of three, healthy donors is shown.

An antigen delivery system should ideally target APC and promote uptake and processing in dendritic cells (DC). Thus, we preliminarily determined whether PS liposome formulation, encapsulated with AF647-BSA, were efficiently internalized by immature and mature human DC. In order to avoid the contribution of free antigen, liposome formulations were dialyzed against a 100 kDa membrane to remove non-encapsulated antigen. Figure 2A shows that the majority of cells became fluorescent after exposure to the liposome formulation, suggesting that most of the cells have taken up the antigen. Moreover, in order to establish whether the antigen was efficiently processed by APC, iDC were exposed to PS liposomes loaded with AF488 BSA and analyzed by confocal microscopy after staining with the acidofilic dye lysotraker red. Results shown in Figure 2B show co-localization of liposomal AF488-BSA with acid compartments. Finally, as acidification of endosomal compartments is a prerequisite for protease activation and the final antigen degradation, we encapsulated PS liposomes with DQ-OVA, which is a fluorogenic substrate for proteases, and monitored fluorescence emission following liposome internalization in iDC. Figure 2C shows 75% of cells exhibiting green fluorescence, as a consequence of proteolytic processing of the protein, and about 23% of cells displaying green/red double fluorescence, indicating endosomal accumulation of the processed antigen. Altogether, these results show that PS/PS liposomes may efficiently deliver the antigen cargo to DC and favor subsequent antigen processing.

### Lipo-AE Enhanced BCG Mediated Protection Against Mtb

**Figure 3:**
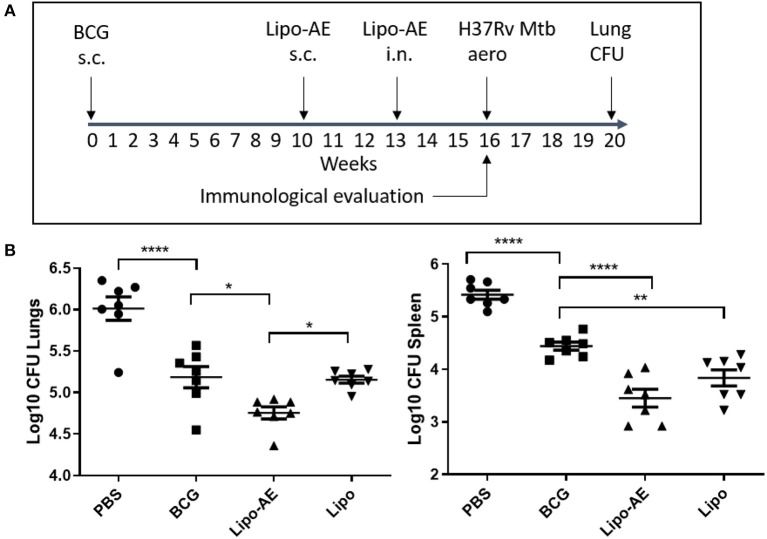
Reduced *Mtb* infection in Lipo-AE immunized mice. Four weeks after the final immunization, mice were challenged with aerosolised *Mtb* and then 4 weeks later culled and organs harvested for bacterial enumeration. Each point corresponds to log CFU value for the individual animals (*n* = 7). **(A)** Schematic depicting immunization and Mtb infection schedule. **(B)** CFU in the lungs and spleens. The horizontal bars represent the mean for each group ± SEM. Log transformed data were analyzed using a 1-way ANOVA and a Dunnett's multiple comparison test comparing all groups to the BCG control or comparing Lipo-AE with empty liposomes (Lipo); *P ≤ 0.05, **P ≤ 0.01, ****P ≤ 0.0001.

In our *Mtb* infection experiment, BCG afforded ~8-fold reduction in the lung and spleen bacterial load (Figure 3), at 4 weeks after the aerosol challenge. Boosting BCG with Lipo-AE imparted additional statistically significant reduction of the bacterial load in both organs sets. The *Mtb* challenge experiment was performed twice and in both instances the Lipo-AE vaccine candidate conferred additional statistically significant protection over BCG alone, though in one of the experiments the non-immunized animals group showed greater than expected intragroup variability (Supplemental Figure S2).

### Lipo-AE Induced Mucosal Antibodies and T Resident Memory Cells (Trm) in Lungs

**Figure 4:**
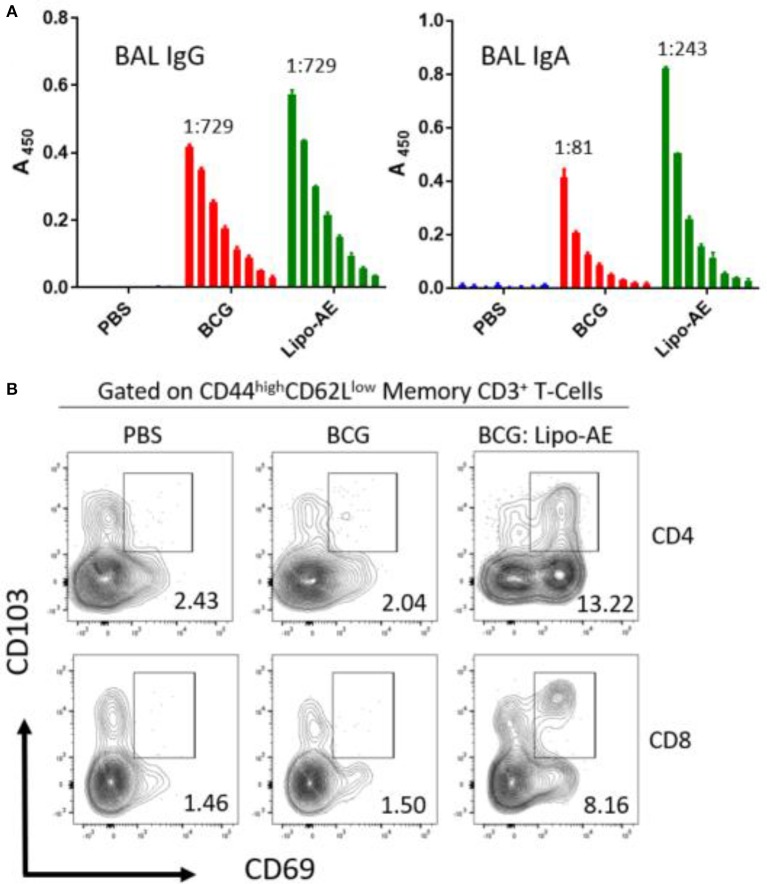
Mucosal antibody responses and lung Trm induced by Lipo-AE. **(A)** Levels of anti-Ag85B specific BAL IgG and IgA determined by antigen-specific ELISA. Each bar corresponds to the mean of triplicate samples from 3 animals from each group ± SEM. The set of bars represent 3-fold serial dilutions from the starting neat sample. Average end point titres are indicated above the bars. **(B)** Analysis of the lung resident memory T cell populations. Except for the PBS group, animals were vaccinated with BCG sc and then with Lipo-AE or empty liposomes, both containing PolyIC, as described in Methods. Cells isolated from the lungs of immunized animals were stained and analyzed by flow cytometry to determine the Trm populations. The gating strategy used was cells -> single cells -> live cells -> CD3+ -> CD4+/CD8+ -> CD44 ^high^/CD62L^low^ and the CD69/CD103 double positive Trm presented. Data originated from *n* = 3 pooled animals and a representative plot is shown.

To measure mucosal responses induced by the vaccine, antibody levels post-immunization were assessed in bronchoalveolar lavage (BAL). BAL collected 3 weeks after final immunization from animals that received Lipo-AE showed similar levels of IgG but higher levels of IgA specific to Ag85B (Figure 4A), compared to BCG immunization alone. No detectable anti-ESAT-6 antibodies were present in either group (not shown).

Upon observing mucosal antibody responses induced by Lipo-AE, we investigated for evidence of cellular immunity. We were particularly interested if there was evidence of T cell resident memory (Trm) in the lungs and used flow cytometry to quantify these cells in the lung homogenates. Prior to harvest, the lungs were perfused to reduce blood contamination. The harvested lungs were then treated and mechanically disrupted to isolate the cells, which were stained for Trm phenotype (Figure 4B). The top and bottom panels represent CD4 and CD8 compartments, respectively. As depicted in the flow plots the total numbers of Trm as defined by the phenotype CD44 ^high^/CD62L ^low^ were very low for the BCG immunized animals, and this was true for both the CD4 and CD8 compartments. In stark contrast, there was a significant increase in Trm populations in the Lipo-AE group for both T cell compartments, increasing from 2.04 in the BCG group to 13.22 in the Lipo-AE groups for CD4+ and from 1.50 to 8.16 for CD8+ T cells, respectively (Figure 4B). Naïve animals showed only background levels of Trm. Although our analysis was restricted to total and not antigen-specific Trm in the lungs, together with the evidence of specific antibodies to Ag85B, it indicates the presence of a mucosal immune response in the lungs of immunized animals.

### Splenic T Cell Proliferation and Polyfunctional T Cells

**Figure 5:**
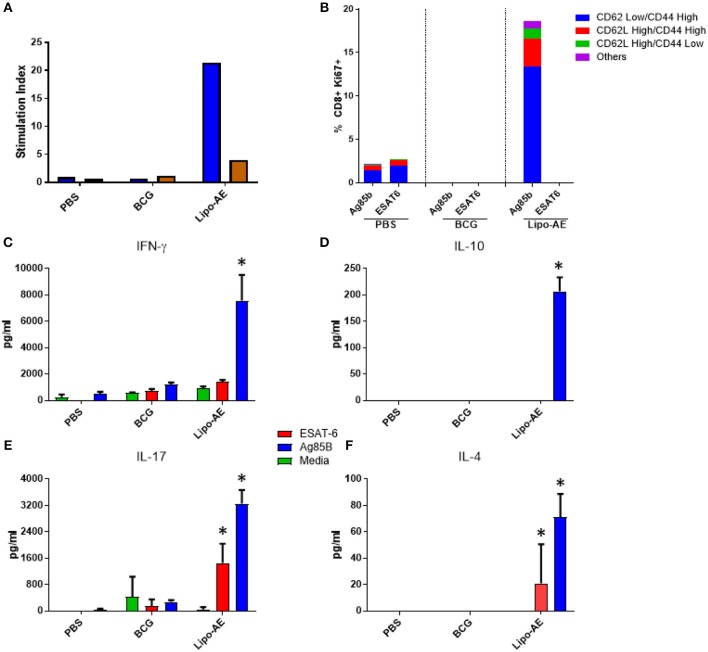
Cellular immune response induced by Lipo-AE. **(A)** Antigen specific splenocyte and T cell proliferation. Splenocytes harvested from immunized animals were stimulated with Ag85B (blue) o ESAT-6 (red) as the recall antigens and media alone as the negative control, and proliferative responses were then measured by radiolabelled thymidine incorporation. Vertical bars represent the stimulation indices (stimulation index = antigen specific radioactive counts per minute/background radioactive counts per minute). **(B)** CD8 T cell proliferation. Splenocytes were stimulated with the recall antigens and the CD8+ Ki67+ cells identified. The gating strategy used was cells -> single cells -> live cells -> CD90.2+ -> CD8+ -> KI67+ and CD44/CD62L cells. The bars represent the percent of CD8+ cells that are Ki67+ and are broken down based on memory marker phenotype. **(D,E,F)** Cytokine production following antigen recall assay. Splenocyte stimulation culture supernatants were collected and assayed for cytokine presence by. Legendplex: IFN-y **(C)**, IL-10 **(D)**, IL-17 **(E)**, and IL-4 **(F)**. Bars depict means pg/ml ± SEM, with *indicating statistically significant values above the background (*P* < 0.05).

We then tested splenocyte proliferation in response to recall antigens as measured by incorporation of radiolabelled thymidine. As shown in Figure 5A, splenocytes from Lipo-AE immunized animals proliferated robustly in response to Ag85B, with a stimulation index of 22, but only modest level of proliferation was observed for ESAT-6, with a stimulation index of 4. Cells from the PBS and BCG groups did not proliferate in response to any of the stimuli. Since liposomes are thought to enhance cross priming and elicit CD8+ T cell responses, T cell proliferation in the CD8+ compartment was additionally investigated by the measurement of Ki67+ cells. Stimulation with Ag85B but not ESAT-6 induced high levels of proliferation in the CD8+ T cell population with most of the proliferating cells being of the CD62L^low^/ CD44^High^ phenotype (Figure 5B). Furthermore, since Lipo-AE induced T cell proliferation (Figure 5A), we speculated that T cell-associated cytokines were also being elicited. To test this, splenocyte culture supernatants were analyzed with a beads-based multiplex immunoassay. The analytes tested were IFN-y (Figure 5C), IL-10 (Figure 5D), IL-17 (Figure 5E), and IL-4 (Figure 5F). Stimulation with Ag85B as a recall antigen induced high levels of all cytokines, reflecting a mixed T cell response with the production of Th1 (IFN-y), Th2 (IL-4), and a Treg cytokines (IL-10 and IL-17), with the latter characteristic of Th17 responses. BCG vaccination resulted in minimal cytokine production in these assays.

**Figure 6:**
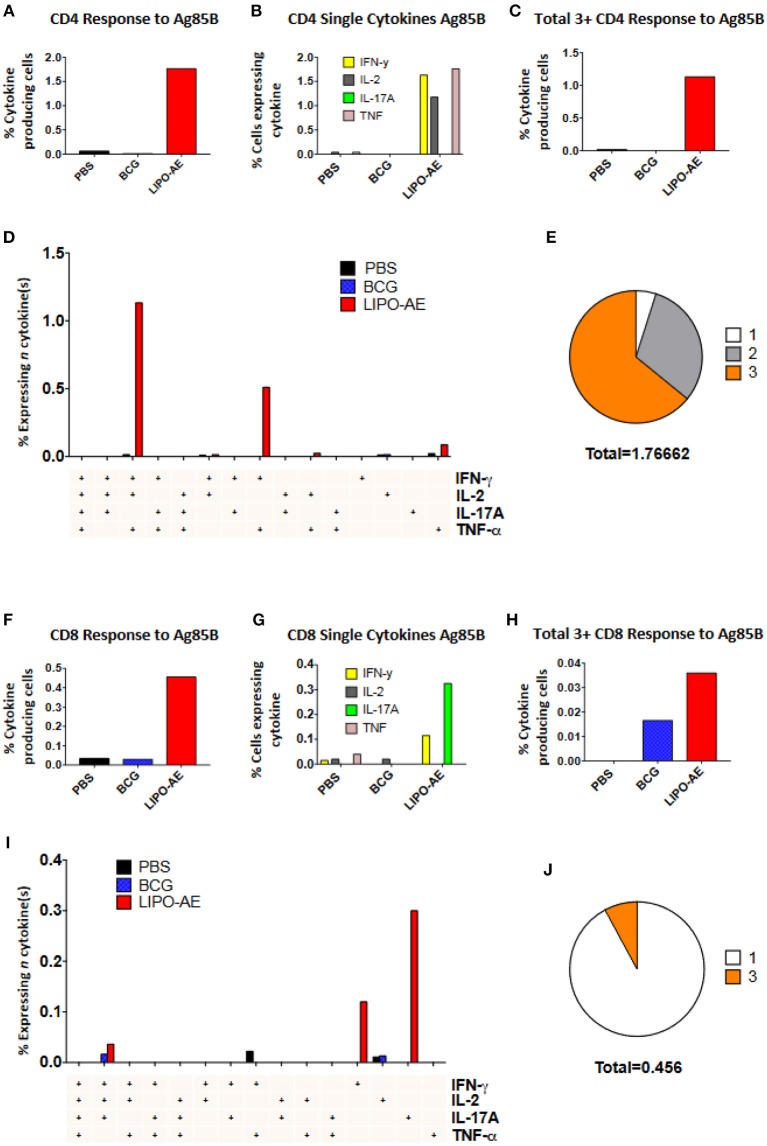
Polyfunctional T cell responses induced by Lipo-AE. Splenocytes isolated from immunized animals were stimulated with Ag85B and the production of IFN-γ, IL-2, IL-17A, and TNF-α measured by intracellular cytokine staining and flow cytometry. **(A,F)** Proportion of CD4 and CD8 positive cells producing cytokines. **(B,G)** Proportion of CD4 and CD8 cells expressing each individual cytokine. **(C,H)** Proportion of CD4 abd CD8 cells producing at least 3 or 4 cytokines. **(D,I)** Polyfunctional CD4 and CD8 T cells phenotype by cytokine combinations. **(E,J)** Frequency of single or multiple cytokine producing CD4 and CD8 T cells as a proportion of all antigen specific cells. Gating strategy was the same as in our recent report (20).

Splenocytes from Lipo-AE immunized animals displayed low level ESAT-6 responses (not shown) but stimulation with Ag85B resulted in a significant number of cytokine producing CD4+ (1.77%) and CD8+ (0.45%) T cells (Figures 6A,F). In the CD4+ T cell compartment, 1.63% of those cells produced IFN-γ, 1.17% IL-2, and 1.76% TNF-α (Figure 6B). Lipo-AE also induced the highest number of polyfunctional T cells producing three or more of the measured cytokines simultaneously with 1.13% of cells in this category (Figure 6C). A large portion of these cells (1.13%) were triple producers, producing INF-γ, IL-2, and TNF-α simultaneously, with a further population (0.51%) double producers of IFN-γ and TNF-α (Figure 6D). Of the cytokine producing cells, most were triple producers (64%) with 31% producing two cytokines and 5% producing only one of the measured cytokines (Figure 6E).

**Figure 7:**
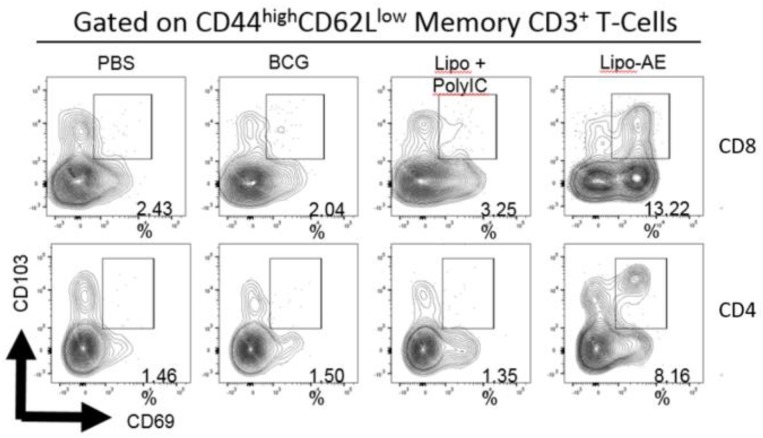
Analysis of the lung resident memory T cell populations. Except for the PBS group, animals were vaccinated with BCG sc and then received one s.c. and one i.n. administration of Lipo-AE or empty PS/PS liposomes with PolyIC. Cells isolated from the lungs of immunized animals were stained and analyzed by flow cytometry to determine the Trm populations. The gating strategy used was cells -> single cells -> live cells -> CD3+ -> CD4+/CD8+ -> CD44 ^high^ / CD62L^low^ and the CD69/CD103 double positive Trm presented. Data originated from *n* = 3 pooled animals and a representative plot is shown.

A similar picture was observed in the CD8+ T cell compartment, where Lipo-AE group splenocytes had the highest number of cytokine producing cells, with 0.11% producing IFN-γ and 0.32% producing IL-17A. The highest number of polyfunctional cells producing 3 or more cytokines simultaneously was also found in the Lipo-AE group (0.036%).

Stimulation with Ag85B resulted in 0.04% of CD8+ T cells producing IFN-γ, IL-2, and IL-17A, with high levels of single cytokine producing cells for IFN-γ, 0.12%, and IL-17A, 0.30%. Of the cytokine producing cells the majority were single producers (92%) with a small proportion of triple producers (8%).

## Discussion

Liposomes have been tested in the context of systemic immunizations but also as oral vaccine vehicles (22, 23). Despite their relative instability, liposomes were shown to be robust enough to survive oral delivery to the intestinal lumen and enhance the antigen presentation (24). Indeed, our own stability experiments showed that the liposomes used in this study are stable for at least 30 days. Subsequent Zetasizer analysis also demonstrated that the formulation was still homogeneous and the size of the liposomes remained unchanged at ~200 nm.

The mucosal delivery of liposomes *in vivo* has been tested extensively through the oral route (22, 25) and also through the intranasal route (26). A comparison between the two routes in terms of ability to induce long term local immune responses seemed to favor the intranasal route (24). Here we demonstrate the ability of a novel liposome formulation to protect against *Mtb* infection when delivered first subcutaneously and then intranasally. This vaccine strategy was explored as a heterologous immunization approach on a BCG background. In human population, boosting BCG would make use of the large number of already immunized individuals whilst taking advantage of protective properties of BCG against severe forms of childhood TB.

Immunization of mice with BCG reduced the burden of infection significantly in the lungs and spleens in comparison to unvaccinated animals in a low dose aerosol challenge model. This protection afforded by BCG was further increased by boosting with Lipo-AE and this added protection was found to be statistically significant in both the lungs and spleens. Upon observing enhancement of protection conferred by Lipo-AE the immunological profile induced by the vaccine was studied. Other pre-clinical studies demonstrated that liposomes promoted antibody and cell mediated immunity to a wide range of bacterial, protozoan, and viral antigens as well as tumor cell antigens, venoms and allergens and even live or attenuated microbial vaccines (6). With regards to cellular immunity, we observed that stimulation of splenocytes from vaccinated animals with a recall antigen induced high levels of IFN-y. IgG and IgA specific to Ag85B was also detected by ELISA in BAL indicating the vaccine had also successfully primed the B cells in the mucosa.

With the view of elucidating further what immune mechanisms could be behind the observed protection we further investigated the cellular profiles induced. A cell proliferation assay revealed that splenocytes isolated from Lipo-AE vaccinated animals in culture proliferated in response to Ag85B and to a lesser extent to ESAT-6. These cells produced high amounts of IFN-y in response to Ag85B stimulation. IFN-y is considered essential for resistance to tuberculosis infection (27, 28). However, despite IFN-y being required for protection a number of studies have suggested that other mechanisms may also be needed for protection (29) and the magnitude of IFN-y response alone is not a reliable correlate of protection (30, 31).

Splenocytes were also found to produce IL-10, which has an ambiguous role in TB, with some reports showing that it is undesirable (32) while other suggesting that it can convert human DCs into macrophage like cells that have increased antibacterial activity against *Mtb* (33). Intriguingly, high levels of IL-17A were also detected in response to Ag85B and to a lesser extent to ESAT-6 in splenocyte cultures from Lipo-AE vaccinate animals. Whilst the role of IL-17 in *Mtb* infection is not clearly defined it has been reported that its induction after vaccination could be beneficial. Thus, Khader et al. hypothesized that IL-17 producing CD4+ T cells occupy the lung after infection and elicit the production of chemokines that attract IFN-y secreting CD4+ T cells resulting in better control of infection (34).

Thus, the cytokine profile characterized by high IFN-y and IL-17A with low levels of IL-10 observed here could be the key to enhanced protection. IL-10 produced by vaccine-induced Tregs could limit collateral damage within the lung. It was suggested that granulomas depended on a balance of inflammatory and anti-inflammatory cytokines in order to effectively control bacteria (35). This concept was validated in the Rhesus macaque model, where sterile granulomas were associated with a balance of IFN-y/IL-17A and IL-10 in contrast to the non-sterile granulomas that featured a predominantly inflammatory response lacking IL-10 (36). Finally, evidence from *T. gondii* model of infection has shown that multifunctional IL-10-producing Th1 cells retain their ability to activate intracellular killing mechanisms in macrophages, and in fact are superior to conventional IL-10 negative Th1 cells at inducing macrophage nitrite (NO) production (37). Given that we observed modest IL-10 and even lower IL-4 production alongside potent Th1/Th17 responses, we therefore conclude that the vaccine induced a strong Th1-Th17 response with only modest Th2-Treg activity and this may have contributed to the protection observed.

The superior immunogenicity of Ag85B over ESAT-6 has also been observed in other studies where both antigens were used together (38, 39), Ag85B is known to be one of the most immunogenic TB antigens whilst ESAT-6 is weakly immunogenic. Nevertheless, both antigens have been previously shown to be protective, particularly when used in combination (38, 39).

Ichihashi et al showed that PS could deliver antigens to APCs resulting in the stimulation of helper and cytotoxic T cell responses *in vivo* (17). In keeping with those observations, a flow cytometry assay measuring Ki67 expression indicated that the CD8 T cells from the Lipo-AE group proliferated robustly in response to Ag85B antigen and had a CD62L^Low^/CD44^High^ phenotype.

CD4+ T cell responses to aerosol *Mtb* challenge are characterized by a delayed recruitment of effector T cells in the lungs, potentially hampering the host's response to infection and permitting the bacteria to establish a persistent infection (40). The rationale behind the heterologous systemic prime mucosal boost strategy was to elicit a robust systemic response with a strong mucosal component to challenge the pathogen on entry. Recently, tissue-memory resident T (Trm) cells have become topical in the field. Thus, Perdomo et al demonstrated that mucosal but not subcutaneous, BCG immunization generates lung resident memory T cell populations that mediate protection against TB (41). We established that Lipo-AE given mucosally could generate Trm, characterized as CD69+CD103+ memory T cells in the lung parenchyma. These cells were found to belong to both CD4 and CD8 compartment and are likely to be antigen specific, though we have no formal evidence of their antigen specificity as we did not have appropriate MHC tetramers to confirm this.

In conclusion, boosting BCG with Lipo-AE mediated enhanced immunity and protection over that afforded by BCG alone. This vaccine delivery platform is well-suited for TB but the liposomal system can be adapted to suit other diseases and recently the first ever liposomes-adjuvanted vaccine was licensed indicated for malaria (42, 43), while a therapeutic vaccine candidate RUTI formulated in liposomes is currently in clinical trials (44). This underscores the potential of liposome technology as an attractive vaccine delivery system.

## Data Availability

The datasets generated for this study are available on request to the corresponding author.

## Ethics Statement

This study was carried out in accordance with the recommendations of UK Home office and St. George's Ethics committee, The protocol was approved by the St. George's University of London.

## Author Contributions

GD, AC, and PH performed all immunization and Mtb infection experiments, as well as key immunological analyses. M-YK performed bronchoalveolar antibody analysis. AT performed statistical analysis. NP generated Lipo-AE and performed APC experiments. MP contributed to processing of tissues and setting up CFUs. MS provided antigens. MF conceived the potential use of liposomes as a vaccine delivery platform. GD, MF, and RR co-wrote the manuscript.

### Conflict of Interest Statement

MS was employed by company Lionex GmBH. The remaining authors declare that the research was conducted in the absence of any commercial or financial relationships that could be construed as a potential conflict of interest.
