# Supplementary material for: Immunization With Mycobacterium tuberculosis Antigens Encapsulated in Phosphatidylserine Liposomes Improves Protection Afforded by BCG
Source: Front Immunol. 2019 Jun 12;10:1349. doi: 10.3389/fimmu.2019.01349 (PMC6598733; doi:10.3389/fimmu.2019.01349)
Supplement: Supplementary file 1 [file Data_Sheet_1.docx]

**Supplemental information**

**Immunization with *Mycobacterium tuberculosis* antigens encapsulated in phosphatidylserine liposomes improves protection afforded by BCG**

Gil R. Diogo^1#^; Peter Hart^1#;^ Alastair Copland^1#^; Mi-Young Kim^1^, Andy C. Tran^1^; Noemi Poerio^3^, Mahavir Singh^2^; Matthew J. Paul^1^, Maurizio Fraziano^3^ and Rajko Reljic^1*^

^1^ St George’s University of London, SW17 0RE, United Kingdom

^2^ Lionex, Braunschweig, 38126, Germany

^3^ University of Rome Tor Vergata, Rome, 00133 Roma, Italy

# These authors contributed equally to this work

* Corresponding author: Dr Rajko Reljic

**Fig.S1**


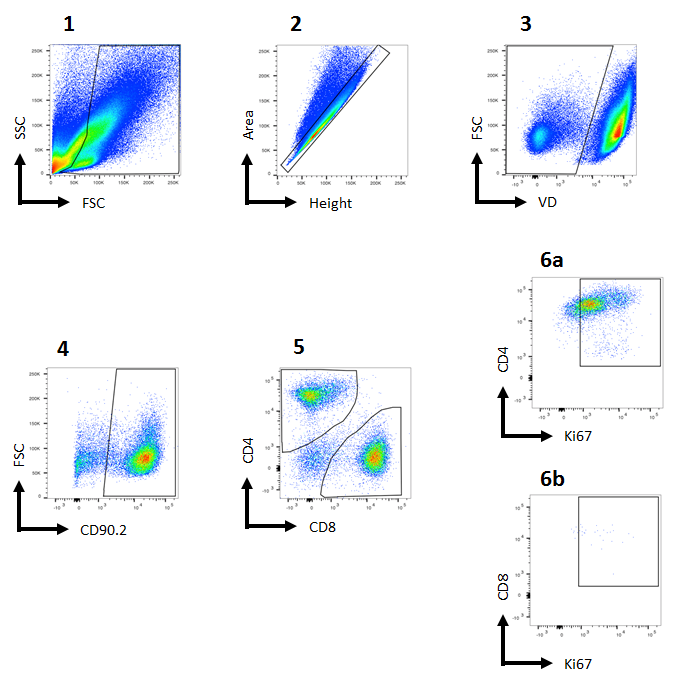


**Supplemental Figure S1. Gating strategy for analysis of T cell proliferation in the splenocyte cultures of LIPO-AE immunised mice.**

**Fig.S2**

**Supplemental Figure S2. Reduced Mtb infection in LIPO-AE immunised mice.** Mice were challenged with aerosolised Mtb and 4 weeks later culled and organs harvested for bacterial enumeration. Each point corresponds to log CFU value for the lungs and spleens of individual animals. (*n* = 5-7; some animals in the lung analysis were omitted due to tissue/plate fungal contamination). The horizontal bars represent the mean for each group ± SEM. Log transformed data were analysed using a 1-way ANOVA and a Dunnett’s multiple comparison test; * P ≤ 0.05, ** P ≤ 0.01.
